# Overcoming ABCB1 mediated multidrug resistance in castration resistant prostate cancer

**DOI:** 10.1038/s41419-024-06949-3

**Published:** 2024-08-01

**Authors:** Sadia Sarwar, Viacheslav M. Morozov, Mallory A. Newcomb, Bowen Yan, Jason O. Brant, Rene Opavsky, Olga A. Guryanova, Alexander M. Ishov

**Affiliations:** 1 Department of Anatomy and Cell Biology, University of Florida College of Medicine, Gainesville, FL USA; 2 Department of Pharmacology and Therapeutics, University of Florida College of Medicine, Gainesville, FL USA; 3 Department of Biostatistics, University of Florida College of Medicine, Gainesville, FL USA; 4 University of Florida Health Cancer Center, Gainesville, FL USA

**Keywords:** Chemotherapy, Cancer therapeutic resistance

## Abstract

Prostate cancer (PCa) is the second leading cause of cancer-related death in American men. PCa that relapses after hormonal therapies, referred to as castration resistant PCa (CRPC), often presents with metastases (mCRPC) that are the major cause of mortality. The few available therapies for mCRPC patients include taxanes docetaxel (DTX) and cabazitaxel (CBZ). However, development of resistance limits their clinical use. Mechanistically, resistance arises through upregulation of multidrug resistance (MDR) proteins such as MDR1/ABCB1, making ABCB1 an attractive therapeutic target. Yet, ABCB1 inhibitors failed to be clinically useful due to low specificity and toxicity issues. To study taxanes resistance, we produced CBZ resistant C4-2B cells (RC4-2B) and documented resistance to both CBZ and DTX in cell culture and in 3D prostaspheres settings. RNAseq identified increased expression of *ABCB1* in RC4-2B, that was confirmed by immunoblotting and immunofluorescent analysis. ABCB1-specific inhibitor elacridar reversed CBZ and DTX resistance in RC4-2B cells, confirming ABCB1-mediated resistance mechanism. In a cell-based screen using a curated library of cytotoxic drugs, we found that DNA damaging compounds Camptothecin (CPT) and Cytarabine (Ara-C) overcame resistance as seen by similar cytotoxicity in parental C4-2B and resistant RC4-2B. Further, these compounds were cytotoxic to multiple PC cells resistant to taxanes with high *ABCB1* expression and, therefore, can be used to conquer the acquired resistance to taxanes in PCa. Finally, inhibition of cyclin-dependent kinases 4/6 (CDK4/6) with small molecule inhibitors (CDK4/6i) potentiated cytotoxic effect of CPT or Ara-C in both parental and resistant cells. Overall, our findings indicate that DNA damaging agents CPT and Ara-C alone or in combination with CDK4/6i can be suggested as a new treatment regimen in CRPC patients, including those that are resistant to taxanes.

## Introduction

Prostate cancer (PCa) is the second leading cause of cancer mortality in American men [1]. PCa that relapses after hormonal therapies (that prevent activation of the main driver of PCa, androgen receptor [AR]), is referred to as castration resistant PCa (CRPC) [2]. CRPC is the cause of almost all PCa-related deaths in the U.S. and often presents with metastases (mCRPC). The few available therapies for mCRPC patients include taxanes docetaxel (DTX) and cabazitaxel (CBZ) [3–5] that remain a mainstay in the clinical practice [6].

Chemotherapy using taxane DTX was the first treatment shown to extend survival in CRPC patients [7]. Most combination therapy regimens in the metastatic castration-naïve and CRPC setting contain DTX (NCCN guidelines 2023, www.nccn.org). In 2010, the FDA approved CBZ, a semi-synthetic taxane derivative, for men with mCRPC previously treated with DTX, or for patients with DTX intolerance (https://www.accessdata.fda.gov/drugsatfda_docs/label/2010/201023lbl.pdf). The most common practice for CRPC treatment is using mitoxantrone-steroid or DTX-prednisone combination as chemotherapy approaches and abiraterone and enzalutamide as hormone control approaches for the first-line treatment, followed by the second-line therapies like CBZ or radium-233 dichloride [8]. CBZ exerts clinical response in mCRPC patients refractory to DTX [9]. This had motivated development of new approaches, such as the combination of taxanes with other drugs. CBZ combinations with known beneficial drugs, such as AR antagonists or radium-223, have the potential to benefit mCRPC patients [10]. Drug combination approaches are currently being tested in 46 out of 86 clinical trials using CBZ (https://clinicaltrials.gov). Nonetheless, clinical success of DTX and CBZ in mCRPC is limited by overt toxicity [11] as well as by high intrinsic and acquired resistance rates [12, 13]. Only ∼50% of CRPC patients respond to taxanes treatment, and all patients eventually fail treatment due to resistance [14]. As a result, each year thousands of men who are treated with taxanes do not benefit from the therapy. Thus, strategies to overcome resistance to taxanes represent the pressing unmet need for the successful treatment of CRPC.

Resistance to taxanes has multiple underlying mechanisms that were extensively characterized [15]. Several of these mechanisms are related to the deregulated mitotic checkpoints and to pathways controlling androgen receptor (AR) signaling and cell survival in stress conditions. Taxanes induce hyper-polymerization of microtubules that activates Spindle Assembly Checkpoint (SAC) [16, 17] and leads to mitotic arrest that eventually triggers cell death [18] by multiple mechanisms [19–22]. Cells resistant to taxanes remain in mitotic block and resume mitosis after drug decay, whereas sensitive cells can react to the extended mitotic block by two complementary mechanisms: apoptosis or mitotic slippage. Apoptosis is one of the outcomes of extensive mitotic block (as described by the Dixit group [23]). It is activated by degradation of anti-apoptotic protein MCL1 by the tumor-suppressor protein FBW7, substrate-binding component of E3 ubiquitin ligase complex. The same group demonstrated that FBW7 is deregulated in multiple tumors and cell lines and is associated with reduced taxane-induced apoptosis. Alternatively, sensitive cells can exit mitosis by a mechanism known as “mitotic slippage” [24, 25], an event biochemically characterized by slow and steady degradation of cyclin B by the E3 ubiquitin ligase, Anaphase Promoting Complex/Cyclosome (APC/C) [24, 26, 27]. The mechanism of APC/C activation in the presence of an active SAC is not well understood. It is well established, however, that as soon as levels of cyclin B (among other APC/C substrates) drop below a threshold, cells exit mitosis in an aberrant micronucleated (MN) G1 stage, a morphological marker of mitotic catastrophe [28, 29]. MN cells often fail the next round of cell division by undergoing apoptosis, necrosis or senescence [30–32] (reviewed in ref. [33]).

The complementary mechanism of taxanes resistance is multidrug resistance (MDR), mediated by upregulation of MDR proteins (MDRPs), members of the family of adenosine triphosphate-binding cassette (ABC)-transporters. MDRPs, as P-glycoprotein MDR1/ABCB1, function as pumps to excrete small molecule toxins including chemotherapeutic drugs out of cells, therefore reducing their bioavailabilty [34]. ABCB1 can transport a variety of substrates out of cells via ATP-hydrolysis including a broad range of molecules, including multiple anti-cancer drugs, such as vincristine, actinomycin-D, paclitaxel, CBZ and DTX [34–36]. Expression of *ABCB1* is elevated in various cancers, including acute myeloid leukemia [37], ovarian carcinoma [38], non-small cell lung cancer [39], breast [40] and PCa [41, 42]. *ABCB1* overexpression is associated with chemotherapy resistance and is elevated after chemotherapy application in several cancers (review in ref. [43]). *ABCB1* expression is induced primarily upon taxane-based treatment [44]. Recent study identified low levels of ABCB1 expression in PCa tissue samples from chemotherapy-naïve patients. Together with post-taxanes therapy induction of ABCB1 [44] (that is also observed in taxane-resistant PC cells [45]), it suggests acquired rather than intrinsic resistance.

Overexpression of *ABCB1* mediates resistance to both DTX and CBZ in CRPC cells [13, 46]. Analysis of ABCB1 levels in chemo-naïve PCa patients has not demonstrated high levels of expression [47], yet it is elevated in PC and is associated with more advanced PC by Gleason score [48]. In addition, levels of ABCB1 are elevated in post-treatment patients PC samples and in multiple PC cell lines, suggesting that ABCB1 upregulation is a result of acquired resistance [42, 45].

In this study, we aimed to identify chemotherapies that can overcome ABCB1-dependent taxanes resistance. Using a cell-based screening platform in conjunction with a curated library of cytotoxic drugs, we found that the DNA damaging compounds camptothecin (CPT) and cytarabine (Ara-C) were cytotoxic to multiple mCRPC cells that overexpressed ABCB1 and had acquired taxanes resistance. In addition, we observed that inhibitors of cyclin-dependent kinases 4/6 (CDK4/6) significantly potentiate cytotoxic effect of DNA damaging drugs. Our results imply that this treatment approach may overcome taxanes resistance and prospectively prevent the development of acquired resistance to taxanes in mCRPC patients.

## Materials and methods

### Cell lines and antibodies

Taxanes-resistant cells (TaxR: DTX-resistant C4-2B, DTXR: DTX-resistant DU145, CTXR: CBZ/DTX-resistant DTXR [49] [were kindly provided by Prof. Allen Gao, University of California, Davis], RC4-2B (CBZ-resistant, production described below), and parental DU145 (ATCC) and C4-2B (ATCC) cells were cultured in RPMI 1640 medium with L-glutamine (Corning #10-040-CV) supplemented with 10% fetal bovine serum (Thermo Fisher Scientific #10437-036) and penicillin/streptomycin (Corning #30-002-Cl) in a humidified incubator at 37 °C with 5% CO_2_. All cell lines were tested for mycoplasma contamination. The following antibodies were used in this study: ABCB1 mouse monoclonal antibody (MDR1 D-11, Santa Cruz #SC-55510), PARP-1 mouse monoclonal antibody (Santa Cruz # sc-8007), Actin mouse monoclonal antibody (Sigma, # A5316), pRb mouse monoclonal antibody (Cell Signaling # 9309), and phospho-pRbS807/811 rabbit monoclonal antibody (Cell Signaling # 8516).

### Colony formation assay and drug treatment

5 × 10^3^ cells were seeded in 12 well plates and treated 36 h later with the following drugs: CBZ (C-2581 LC Laboratories), DTX (D-1000, LC Laboratories), Camptothecin (CPT, 11694, Cayman Chemicals), Cytarabine (Ara-C, 16069, Cayman Chemicals), Doxorubicin (Dox, D-4000, LC Laboratories), Irinotecan (CPT-11; I-4122, LC Laboratories), ABCB1i Elacridar (# S7772, Selleck); CDK4/6 inhibitors: Palbociclib (P-7788, LC Laboratories), Ribociclib (R-8200, LC Laboratories), Abemaciclib (S5716, Selleckhem) according to protocols specified in individual experiments for the indicated time. Cells were fixed 9 days after seeding for 10 min with 4% formaldehyde and stained with crystal violet (0.5%). Images were acquired with Epson photo scanner and area of colonies were calculated using ImageJ software. Experiments were independently repeated at least three times in technical triplicates.

### 3D model (prostaspheres)

The prostaspheres were grown in Matrigel (Corning) supplemented with RPMI 1640 complete media in 24-well plates as described [50]. The basal layer was formed by mixing Matrigel and medium at a ratio 1:1; 250 μl of mixture was added per well. Cells were resuspended in complete media, mixed with Matrigel at a ratio 4:1; 10^3^ cells were added onto pre-solidified base layer. The plate was placed in tissue culture incubator to allow the upper layer to solidify. Next, 1 ml of complete media was added to each well. Prostaspheres were treated with corresponding drugs at day 3 for indicated time. Prostaspheres were stained with Calcein AM (Invitrogen) 21 days post treatment (100 μl of 3.3 mM Calcein AM per well for 20 min in the tissue culture incubator). Prostaspheres were imaged using Leica fluorescent microscope and analyzed using ImageJ software. Experiments were repeated at least three times.

### Prostaspheres processing for colony formation assay

Prostaspheres were re-suspended in 1 ml PBS. After 3 min of centrifugation at 300 RCF, supernatant was aspirated, and cell pellet was resuspended in 0.5 ml of the remaining supernatant. Equal volume of trypsin was added for 3 min. Cells were resuspended in 4 ml RPMI complete media and 500 cells from control prostaspheres were plated per well of 6-well plate in triplicates. The same volume of cell suspension (as used in control) was plated for each treatment condition. Three weeks later, the colonies were fixed, stained with crystal violet, imaged, and analyzed using ImageJ. Experiments were repeated at least three times.

### Alamar blue cell viability assay

Cells (3 × 10^3^) were seeded in 96 well plates, 24 h later treated with drugs as indicated in individual experiments. At the end of treatment, Alamar blue solution (10% volume of media) was added. Fluorescence was measured using Spectra Max M3 plate reader after 4 h of signal development. Experiments were repeated at least three times with six technical replicates in each experiment.

### Analysis of clinical data

Firehose Legacy, Fred Hutchinson CRC [51] and Metastatic Prostate Adenocarcinoma (SU2C/PCF Dream Team [52] datasets were accessed via cBioPortal [53, 54].

### Statistical methods

Statistical analysis was performed with GraphPad Prism 9.2.0 (GraphPad Software, Inc., San Diego, CA) and statistical tests were applied as described in the figure legends.

### RNA-seq

1 × 10^6^ cells were plated on 6 cm dish in RPMI 1640 medium, 10% CSS (Invitrogen), with L-glutamine (Corning #10-040-CV) supplemented with 10% fetal bovine serum (Thermo Fisher Scientific #10437-036) and penicillin/streptomycin (Corning #30-002-Cl) and grown for 72 h. RNA was isolated using RNeasy Plus Kit (QIAGEN, # 74134) according to the manual and sequenced at Novogene.

### RNA-Seq bioinformatic analysis

Short reads were mapped to the GRCh38 reference transcriptome using STAR [55]. Quantification produced tables of FPKM values for each gene in each sample. Differential analysis was performed with DESeq2 [56], generating tables of significantly over- or under-expressed genes in each contrast.

### Immunofluorescence studies

Immunofluorescence was done as described [50]. Briefly, 75 × 10^4^ cells were grown on microscope coverslip glass (Fisher Scientific, Hampton, NH; cat # 1255015) in RPMI1640/10% FBS media. Cells were fixed with 1% formaldehyde for 10 min, permeabilized with 0.5% Triton X-100, and incubated with primary antibodies for 1 h at room temperature. After two washes with PBS, cells were incubated with secondary antibodies conjugated with Alexa Fluor 488 or 594 dye (Invitrogen, Thermo Fisher Scientific, Waltham, MA). DNA was stained with Hoechst 33342 (Sigma, St. Louis, MO). Images were acquired using Leica TCS SP5 confocal microscope.

### Western blotting analysis

Cells were lysed directly in Laemmli sample buffer. Protein samples were separated by 4–20% SDS-PAGE (Bio-Rad, Hercules, CA) and transferred to nitrocellulose membrane using iBlot 2 system (Invitrogen). Membranes were blocked with: 5% nonfat milk/PBS, 0.1% Tween (PBST) for ABCB-1 and PARP-1 primary antibodies, and with 5% BSA/TBS, 0.1% Tween (TBST) for pRb and phospho-pRbS807/811 primary antibodies; either blocking solutions were used for actin primary antibodies. Primary antibodies were diluted in the corresponding blocking solution and incubated overnight at 4 °C. Accordantly, membranes were washed three times with PBST or TBST and incubated for 1 h at ambient temperature with appropriate IRDye secondary antibody (Li-COR Biosciences). Membranes were washed three times with PBST or TBST and visualized by Odyssey CLx Imaging System (Li-COR Biosciences).

### FACS analysis of cell cycle

1 × 10^6^ cells were treated with indicated concentrations of Ribociclib for 24 h. Following treatment, incubation media were collected, cells were trypsinized and combined with media. Cells were centrifuged at 500 rcf for 3 min, the pellet was washed with cold PBS. Cells were resuspended in ice-cold PBS and mixed with equal volume of ice-cold ethanol by pipetting and kept on ice for at least 30 min. After fixation, the cell pellet was resuspended in PBS containing 1 µg/mL DAPI and incubated for 10 min at room temperature, protected from light. Flow cytometric analysis was performed using an LSR Fortessa flow cytometer with Diva software (BD Biosciences). The data were analyzed using FlowJo software (v10.4.1).

## Results

### Production and characterization of taxanes resistant cells

**Fig. 1:**
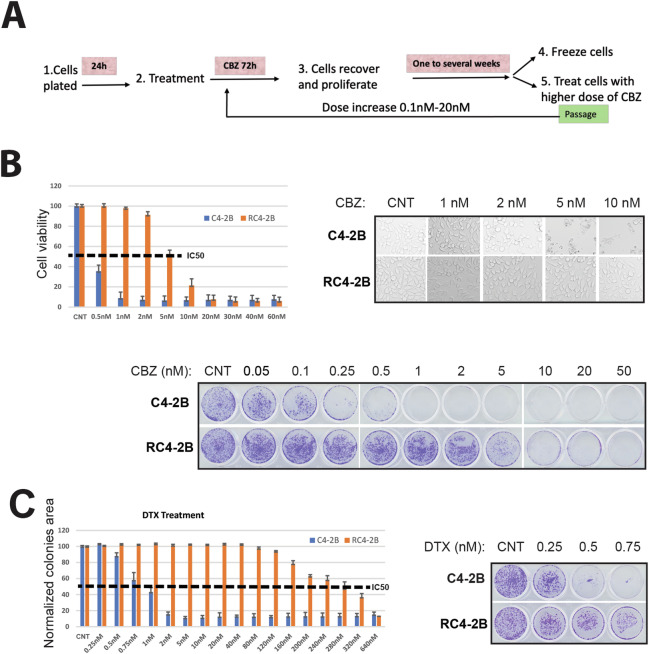
Production and characterization of CBZ-resistant cells. **A** Experimental scheme outlining production of CBZ-resistant cells. CRPC C4-2B cells were plated in 50% confluency and treated with CBZ 24 h later. After 72 h of CBZ treatment, cells were recovered for one to several weeks, re-plated, and cycle was repeated with increased concentration of CBZ ranging from 0.1 to 20 nM. **B** C4-2B (parental) and RC4-2B CBZ-resistant cells were treated with indicated concentrations of CBZ. Results of cell viability assay (left), representative phase contrast images (right) and colony formation assay (bottom). IC50 for CBZ is ~10× higher in RC4-2B cells compared to the parental C4-2B cells. **C** C4-2B and RC4-2B cells were treated with indicated concentrations of taxane DTX. Results of cell viability assay (left) and colony formation assay (right). IC50 for CBZ is ~300× higher in RC4-2B cells compared to the parental C4-2B cells. CNT control.

To study taxanes resistance in CRPC, we have produced CBZ-resistant mCRPC C4-2B cell line following experimental scheme outlined in Fig. 1A. After 72 h of CBZ treatment, cells were recovered for one week, re-plated, and the cycle was repeated with increased concentration of CBZ starting at 0.1 nM and ending at 20 nM. After 4 months, we successfully completed the production of CBZ resistant subclone RC4-2B.

**Fig. 2:**
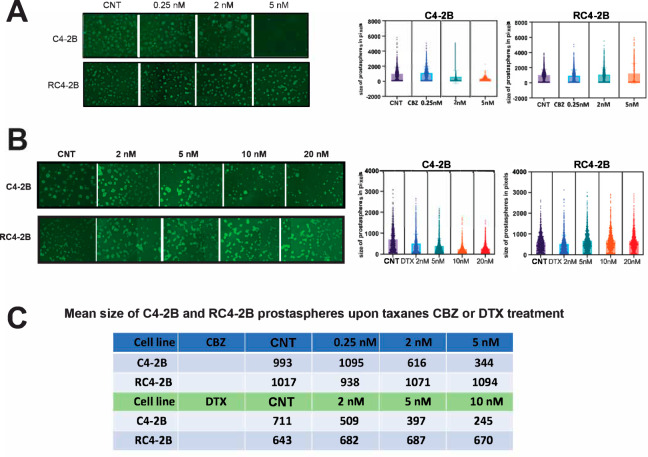
Characterization of CBZ and DTX resistance of RC4-2B cells by 3D prostasphere model. C4-2B (parental) and RC4-2B CBZ-resistant cells were set up in Matrigel and treated at day 3 with indicated concentrations of taxanes CBZ or DTX for 24 h; at day 21, prostaspheres were stained with cell viability marker Calcein AM and documented. Left: representative images of prostaspheres treated with CBZ (**A**) or DTX (**B**). Right: individual area of prostaspheres was analyzed by ImageJ and calculated by Graph PAD Prism. RC4-2B cells are more resistant to CBZ in prostaspheres settings compared with C4-2B parental cells. **C** Table shows mean size of C4-2B and RC4-2B prostaspheres after treatment with CBZ or DTX. CNT control.

We characterized CBZ resistance of RC4-2B cells using metabolic viability (Alamar Blue) and colony formation assays. These complementary methods revealed that IC50 for CBZ is ~10× higher in RC4-2B cells compared to the parental C4-2B cells (Fig. 1B). Taxanes CBZ and DTX have similar mechanism of action determined by tubulin binding, that block microtubule depolymerization. Nonetheless, these drugs have different clinical activity in mCRPC patients, and CBZ is recommended in patients refractory to DTX (NCCN guidelines 2023, www.nccn.org). Thus, we aimed to determine cross-resistance to DTX in CBZ resistant RC4-2B cells. Using proliferation and colony formation assays, we observed that CBZ-resistant RC4-2B cells were also resistant to DTX; IC50 for DTX was ~300× higher in RC4-2B cells compared to the parental C4-2B cells (Fig. 1C). To further characterize taxanes resistance, we used 3D-Matrigel (prostasphere) model [50] combined with direct analysis of cell viability in prostaspheres with the cell viability marker Calcein AM. As in 2D conditions, CBZ and DTX eradicated the parental C4-2B prostaspheres at lower concentrations compared with RC4-2B (Fig. 2).

### MDRP ABCB1 overexpression triggers taxanes resistance in RC4-2B

**Fig. 3:**
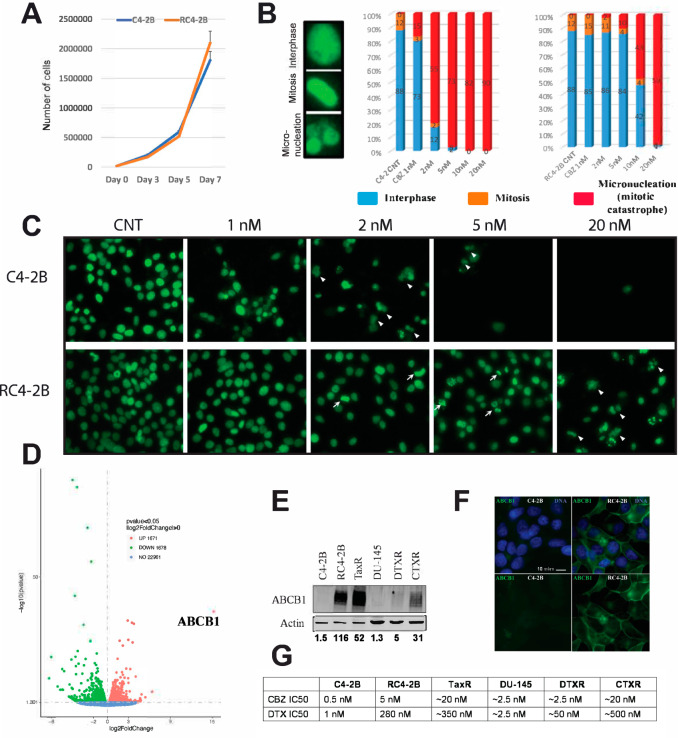
Characterization of taxanes treatment in PC cells. **A** Proliferation (analyzed by cells count in triplicates) is similar in C4-2B and RC4-2B cells. **B**, **C** Morphological analysis of CBZ treatment. Cells were treated for 48 h with indicated concentrations of CBZ and stained with Hoechst (pseudo colored green). **B**, left: representative images of interphase, mitosis, mitotic catastrophe (micronucleated, MN) cells. 5 independent fields (at least 500 cells) were analyzed for their morphology; C4-2B (**B**, middle) and RC4-2B (**B**, right). C4-2B and RC4-2B cells have similar mitotic index (~12%) in control conditions. C4-2B cells activate mitotic catastrophe (result of slippage from the CBZ-induced mitotic block) starting at 1 nM of CBZ. RC4-2B cells are more resistant to induction of both mitotic block and mitotic catastrophe. **C** Representative images after indicated treatment of C4-2B (top) and RC4-2B (bottom). Arrow: mitotic cells; arrowheads: MN cells. **D** Expression analysis (RNAseq, volcano plot) of C4-2B and RC4-2B cells. Numbers in figure legends correspond to genes that are up- (red) or downregulated (green) in RC4-2B cells compared to C4-2B cells (*p*-adjusted < 0.05). X: expression, log2; Y: *P*-value, log10. *ABCB1* expression is significantly upregulated in RC4-2 cells compared to C4-2B cells (log2 = 15.2; *P*-value = 2.8E−37; adjusted *P*-value = 7E−34). **E** Immunoblot analysis of ABCB1 protein levels in: C4-2B (parental) and C4-2B derived RC4-2B (CBZ resistant) and TaxR (DTX-resistant) cells, DU145 (parental) and DU145-derived DTXR (DTX-resistant), CTXR (CBZ/DTX-resistant) cells. ABCB1 is elevated in all four resistant cell lines compared with parental cells. The highest levels of ABCB1 protein were documented in RC4-2B and TaxR, intermediate in CTXR (CBZ/DTX-resistant DU145-DTXR), and low in DU145-DTXR (DTX-resistant DU145). Actin: loading control. Numbers below: the relative levels of ABCB1 normalized to actin. **F** Microscopy analysis of ABCB1 in C4-2B (left) and RC4-2B (right) cells. Top: overlay of ABCB1 (green) and DNA (blue). Bottom: ABCB1 (green). ABSB1 is expressed at the background levels in C4-2B, and is highly expressed in RC4-2B, with expected localization at the plasma membrane. **G** IC50 (determined by Alamar Blue assay) for CBZ and DTX in C4-2B, RC4-2B, TaxR, DU-145, DTXR and CTXR cell lines. Taxanes resistance in these cell lines is proportional to the levels of ABCB1 expression documented in **E**. CNT control.

To address the mechanism of taxanes resistance in RC4-2B cells, we first characterized proliferation rate of CBZ-resistant RC4-2B and parental C4-2B cells, reasoning that reduced proliferation may diminish population of cells undergoing mitosis and thus targeted for taxanes-induced cytotoxicity. Our results showed a similar rate of proliferation (Fig. 3A) and an almost identical mitotic index (12%, Fig. 3B right), arguing that resistance is not due to changes in proliferation rate or in cell cycle distribution between these cell lines. We investigated the cellular response to CBZ using morphological analysis (Fig. 3B left for representative images of interphase, mitosis, and micronucleated [MN] cells). Parental C4-2B cells were blocked in mitosis with increased concentrations of CBZ and exited this block by mitotic catastrophe that was characterized by MN. RC4-2B cells were resistant to mitotic block and continued to proliferate (as indicated by mitotic cells) at CBZ concentrations that were inducing mitotic catastrophe in C4-2B cells (Fig. 3B, C).

**Fig. 4:**
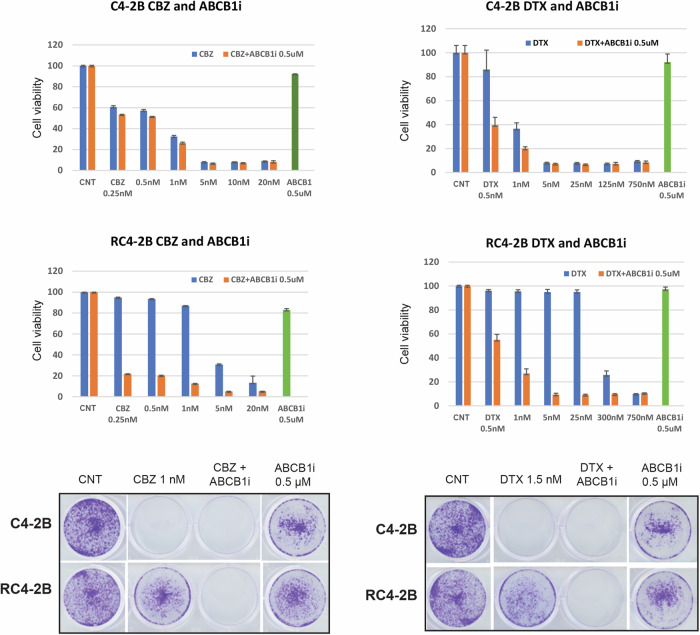
ABCB1-specific inhibitor elacridar reverses CBZ and DTX resistance in RC4-2B cells. C4-2B and RC4-2B CBZ-resistant cells were treated with indicated concentrations of CBZ (left) or DTX (right) alone, in combination with 0.5 uM of ABCB1 specific inhibitor elacridar, or with 0.5 uM of elacridar alone. After 72 h of treatment, survival was analyzed by Alamar Blue (top) or colony formation assays (bottom). Inhibition of ABCB1 reversed resistance to both CBZ and DTX in RC4-2B cells. Treatment by elacridar alone has minimal effect on C4-2B or RC4-2B cells. CNT control.

To further understand mechanisms of taxanes resistance, we performed transcriptome analysis in C4-2B and RC4-2B cells. Expression analysis by RNAseq identified several up- and down-regulated genes in RC4-2B (Volcano plot, Fig. 3D). The most differentially expressed gene was the MDRP *ABCB1*, that was significantly upregulated in RC4-2B compared with C4-2B cells (log2 = 15.2; *P*-value = 2.8E−37, adjusted *P*-value = 7E−34). We confirmed the increased expression of ABCB1 protein by Western blot and by immunofluorescent microscopy analyses (Fig. 3E, F). Upregulation of *ABCB1* in CBZ-resistant RC4-2B cells recapitulates the clinical response of mCRPC patients [42]. To functionally address ABCB1 dependent taxanes resistance, we tested whether ABCB1 inhibition can reverse DTX and CBZ resistance in RC4-2B cells using ABCB1-specific inhibitor elacridar [57, 58]. While elacridar alone had minimal effect on both C4-2B and RC4-2B, it reversed CBZ and DTX resistance in RC4-2B (Fig. 4), confirming ABCB1-dependent resistance mechanism in these cells.

### DNA damaging drugs camptothecin (CPT) and cytarabine (Ara-C) have the same cytotoxicity in parental and taxanes resistant cells

**Fig. 5:**
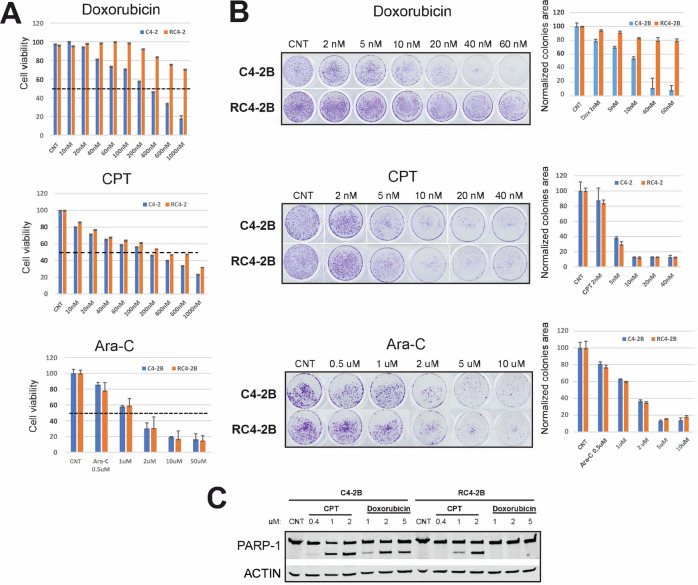
DNA damage drugs camptothecin (CPT) and cytarabine (Ara-C) are active in taxanes resistant RC4-2B cells. C4-2B (parental) and RC4-2B taxanes-resistant cells were treated with indicated concentrations of doxorubicin (Dox, top), CPT (middle) and Ara-C (bottom). Survival (Alamar Blue assay, **A**) and colony formation assay (**B** representative images [left], colonies area analysis [right]). RC4-2B cells are resistant to doxorubicin. CPT and Ara-C eradicated the taxanes-resistant RC4-2B cells at similar concentrations as those for the taxanes-responsive parental C4-2B cells. **C** Western blot analysis of PARP-1 in C4-2B and RC4-2B cells after indicated concentrations of doxorubicin and CPT treatment for 24 h. CPT induces PARP-1 cleavage (marker of apoptosis) at the same concentrations in both cell lines, while doxorubicin is effective only in parental C4-2B cells. Actin: loading control. CNT: control. Dashed lines: IC50.

Screen of chemotherapeutic drugs that are not substrates of ABCB1 may identify avenues to overcome ABCB1-dependent MDR in mCRPC patients, including ABCB1-dependent taxanes resistance. We performed cell-based screen with antitumor drugs comparing cytotoxicity in RC4-2B and C4-2B cells. While RC4-2B cells were resistant to doxorubicin (Fig. 5A, B, Supplementary Fig. S1) and to Irinotecan (Supplementary Fig. S1), we observed that the DNA damaging compounds camptothecin (CPT) and cytarabine (Ara-C) are cytotoxic to RC4-2B. Treatment with either compound eradicated the taxanes-resistant RC4-2B cells at similar concentrations as those for the parental taxanes-responsive C4-2B cells in proliferation (Fig. 5A), colony formation (Fig. 5B) and prostasphere (Supplementary Fig. S1) assays. We did not observe substantial apoptotic death of RC4-2B cells after doxorubicin treatment (tested by cleaved Poly [ADP-ribose] polymerase 1 [PARP-1]), while increasing concentration of CPT induced accumulation of cleaved PARP-1 in both cell lines indicating similar dynamics of apoptosis induction by this DNA damaging agent in both cell lines (Fig. 5C). Thus, we observed that taxanes resistant cells are also resistant to two DNA damaging agents, doxorubicin and Irinotecan, but are sensitive to CPT and Ara-C.

### Characterization of taxanes resistance in additional CRPC cell lines

**Fig. 6:**
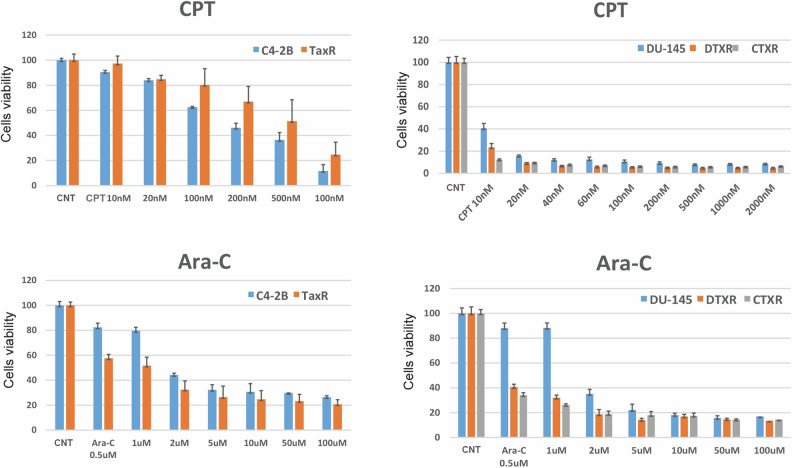
CPT and Ara-C are active in multiple taxanes resistant PC cell lines. C4-2B and DU145 (parental), TaxR (C4-2B derived DTX-resistant), DTXR (DU145 derived DTX resistant), and CTXR (DTXR derived DTX/CBZ resistant) cells were treated with indicated concentrations of CPT or Ara-C. After 72 h, cell survival was measured by Alamar Blue assay. CPT eradicated the taxanes-resistant cells at similar concentrations as those for the taxanes-responsive C4-2B and DU145 cells, and Ara-C is more cytotoxic on taxanes-resistant cells. CNT control.

To confirm our observations that CPT and Ara-C can overcome DTR and CBZ resistance, we tested additional taxanes resistant cell lines, including TaxR (DTX-resistant C4-2B), DTXR (DTX-resistant DU145), and CTXR (CBZ/DTX-resistant DTXR) [49]. All three cell lines overexpress ABCB1 [49] (Fig. 3E), confirming common characteristic of ABCB1-dependent taxanes resistance. In addition, we observed that levels of ABCB1 differed among resistant cells, with the highest levels in RC4-2B and TaxR (CBZ- and DTX-resistant C4-2B), intermediate in CTXR (CBZ/DTX-resistant DTXR), and lowest in DU145-DTXR (DTX-resistant DU145). We confirmed DTX and CBZ resistance in these additional cell lines (Fig. 3G). Taxanes resistance in four tested cell lines was proportional to the levels of ABCB1 expression (compare Western blot results and IC50 for CBZ and DTX, Fig. 3E, G). ABCB1 inhibitor elacridar restored sensitivity to both CBZ and DTX in all three cell lines (Supplementary Fig. S2) similarly to RC4-2B (Fig. 4), confirming common ABCB1-dependent mechanism of taxanes resistance in these cell lines. Finally, we observed that these taxanes-resistant cell lines were equally susceptible to CPT- and Ara-C-induced cell killing as parental C4-2B and DU145 cells (Fig. 6). This further supports the idea that these drugs are active in ABCB1-dependent MDR resistant CRPC cells and, potentially, can overcome this resistance in CRPC patients.

### CDK4/6 inhibition potentiates cytotoxic activity of CPT and Ara-C in parental and taxanes resistant cells

Cyclin-dependent kinase-4 and cyclin-dependent kinase-6 (CDK4 and CDK6) are often overexpressed in cancer, including CRPC, and are considered as targets for cancer treatment [59]. At present, three CDK4/6 inhibitors (CDK4/6i) Palbociclib, Ribociclib and Abemaciclib are FDA-approved and are further evaluated in clinical trials in several cancers, including breast cancer, non-small and small cell lung cancer, and prostate cancer [59]. Therefore, we tested whether CDK4/6i can potentiate the activity of Ara-C and CPT in parental and taxanes-resistant cells.

First, we compared the effect of three CDK4/6i: Palbociclib, Ribociclib and Abemaciclib, on parental C4-2B and resistant RC4-2B cells. We observed similar activity of these inhibitors in both cell lines (Supplementary Fig. S3), indicating that all three tested CDK4/6i were not substrates of ABCB1. We observed similar effect of Ribociclib at the cell cycle inhibition (tested by FACS analysis) and reduction of pRb phosphorylation at serines 807 and 811 (that are substrates of CDK4/6 [60]) in both cell lines (Supplementary Fig. S4). As previously published [61], we observed some reduction of total pRb levels after Ribo treatment.

**Fig. 7:**
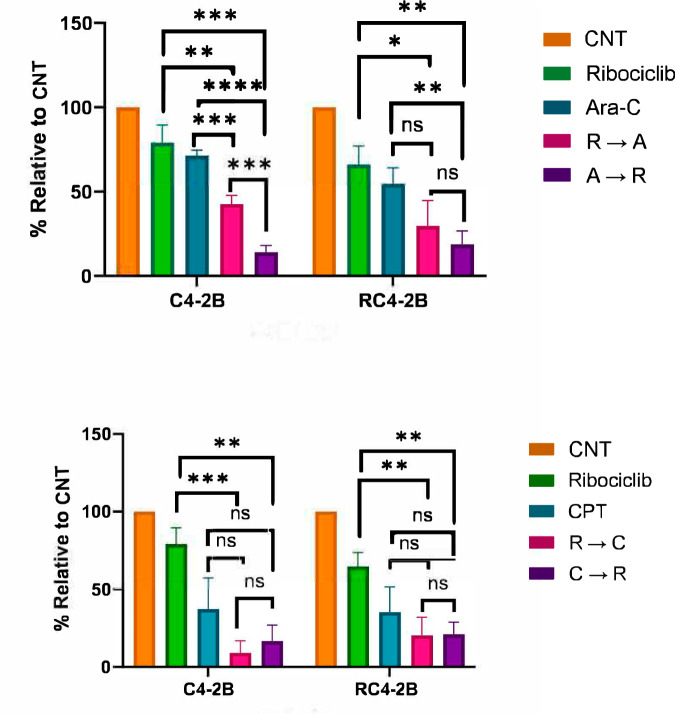
CDK4/6i Ribociclib potentiates cytotoxic activity of Ara-C and CPT in parental and taxane- resistant cells. C4-2B and RC4-2B cells were treated with single agents (Ribociclib [1 uM, 7d], Ara-C [1 uM, 24 h, top], or CPT [6 uM, 24 h, bottom]), with Ribociclib (24 h) followed by Ara-C or CPT (24 h), followed by Ribociclib 5d (R → A or R → C), or by Ara-C or CPT (24 h) followed by Ribociclib 6d (A → R or C → R). Effect was analyzed by colony formation assay and quantified by Image J. The error bars represent the standard devisation of three independent experiments. ^ns^*p* > 0.05; *p ≤ 0.05; **p ≤ 0.01; ***p ≤ 0.001; ****p ≤ 0.0001. CNT control.

Both Ara-C and CPT are active during S-phase, while CDK4/6i Ribociclib is active mostly at the end of G1, preventing S-phase entry. Thus, we tested effect of combined treatment by Ribociclib with Ara-C or CPT in two settings: Ribociclib followed by Ara-C or CPT, or reverse sequence of application, Ara-C or CPT followed by Ribociclib. We observed that Ribociclib potentiates cytotoxic activity of both DNA damaging agents in parental and RC4-2B cells, and that this effect was significant for Ara-C (Fig. 7). In addition, Ara-C followed by Ribociclib had stronger effect compared with reverse, indicating importance of drugs application sequence.

## Discussion

Resistance to taxanes is the most limiting factor for their clinical use in the treatment of mCRPC patients, and overcoming this resistance is expected to reduce patient mortality. Failure of taxanes therapy is often associated with overexpression of multidrug transporter ABCB1, which is acting as an efflux pump that reduces intracellular concentration of chemotherapeutic agents. Overexpression of ABCB1 was documented after doxorubicin treatment in a genetically engineered mouse model (GEMM) of breast cancer, resulting in MDR including DTX [62]. In similar studies, elevation of ABCB1 was documented in GEMM after tumors developed resistance to PARP inhibitor olaparib [63]. Expression of *ABCB1* was significantly increased in the experimentally produced taxanes-resistant CRPC cell lines by several groups (including current study), thereby recapitulating the clinical response of mCRPC patients [13, 45, 46, 64, 65] Mechanisms of ABCB1 overexpression include epigenetic changes at *ABCB1* regulatory elements [66], amplification of the *ABCB1* gene [46], and increased activation of transcription factors that regulate *ABCB1* expression [67].

ABCB1 expression is induced primarily upon taxane-based treatment [44]. Low levels of ABCB1 expression was documented in prostate cancer tissue samples from chemotherapy-naïve patients, suggesting acquired resistance. We performed analysis relating ABCB1 levels with therapy and disease progression (Supplementary Fig. S5). Expression of ABCB1 has high variability between patients; we did not observe significant increase in the ABCB1 expression after taxanes treatment (Supplementary Fig. S5A, analysis of ABCB1 levels in chemotherapy naïve and taxanes treated patients, Metastatic Prostate Adenocarcinoma SU2C/PCF Dream Team dataset [52]). Unfortunately, this dataset does not compare pre- and post-treatment levels of ABCB1; it also does not have data on disease progression after treatment, renders impossible to correlate ABCB1 levels with the treatment outcome. We also analyzed ABCB1 levels relative to the therapy outcome (Supplementary Fig. S5B, Prostate Adenocarcinoma, TCGA, Firehose Legacy [51]). Though none of the groups were statistically significant, higher expression of ABCB1 correlates with the progressive disease in PC patients. Unfortunately, this dataset does not include history of taxanes treatment. Yet, given that the most common practice for CRPC treatment is using DTX in the first-line treatment, followed by the CBZ as the second-line chemotherapies, results suggest function of ABCB1 in treatment response of PC patients. Indeed, future analysis that combines data of pre-and post-taxanes treatment levels of ABCB1 and treatment outcome would provide results to adjust treatment strategies.

To study taxanes resistance in CRPC, we have produced CBZ-resistant mCRPC RC4-2B cell line and confirmed resistance using complementary assays, including in 3D prostaspheres settings. As expected, CBZ resistant cells are also resistant to DTX, yet to much higher levels: IC50 for CBZ is ~10x higher in RC4-2B cells compared to the parental C4-2B cells, while IC50 for DTX is ~300x higher. Our results provide a mechanistically-informed rationale for the clinically approved CBZ use as a second line taxane in mCRPC patients that are refractory to DTX [9]. We found that taxanes resistant RC4-2B cells overexpress MDRP ABCB1, thereby recapitulating the clinical response of mCRPC patients with acquired taxanes resistance. We observed that ABCB1-specific inhibitor elacridar reversed DTX and CBZ resistance in RC4-2B and several additional taxanes resistant mCRPC cell lines, confirming ABCB1 function in therapeutic refractoriness. In addition, we observed that taxanes resistance in four tested CRPC cell lines was proportional to the levels of ABCB1 expression (Fig. 3E, G), suggesting treatment options (such as escalation of taxanes doses) depending on the levels of ABCB1 expression in individual CRPC patients, toxicity permitting.

There are several ways to overcome ABCB1-dependent MDR. First, extensive research is ongoing to design new ABCB1 inhibitors [68] and to repurpose FDA approved drugs that have ABCB1 inhibitor activity [45, 68]. Given that MDR is common in chemotherapy resistance, efforts have been directed towards the development of new P-glycoprotein inhibitors, such as elacridar, zosuquidar, laniquidar (R101933) and tariquidar (XR9576) [68]. These drugs have demonstrated high potential to reverse taxanes resistance in experimental models but, unfortunately, had minimal effects in Phase II clinical trials due to low specificity and toxicity issues [69–71]. Second, studies are ongoing to identify cancer-specific regulators of ABCB1 expression for their potential targeted deactivation [67]. Finally, screens to identify chemotherapeutic drugs that are not substrates to ABCB1 and, therefore, are not excreted by ABCB1, offer promise to identify mediators and tools to overcome ABCB1-dependent taxanes resistance in cell models, that can be further developed for clinical applications. To this end, recent studies have identified nucleotide analogue gemcitabine [72] and antifungal drug itraconazole [45] as active cytotoxic agents in taxanes-resistant PC cells.

DNA damage response pathways, including inhibition of PARP, are emerging targets in PC treatment. Unfortunately, most of these treatment demonstrate only marginal increase in patients survival due to multiple reasons, including deregulation of DNA damage response pathways [73]. In addition, MDR and, specifically, *ABCB1* overexpression, renders cancer cells, including CRPC cells, resistant to PARP inhibitor Olaparib [49, 74, 75]. Moreover, *ABCB1* overexpression determines Olaparib resistance in ovarian cancer patients [76]. Therefore, there are urgent needs to identify new treatment regimen in CRPC patients, including those that are resistant to taxanes due to ABCB1 elevation.

Using a cell-based screen with antitumor drugs, we observed that taxanes resistant RC4-2B cells were also cross-resistant to DNA damaging compounds doxorubicin and Irinotecan (a water-soluble Camptothecin derivative), indicating that those compounds are likely substrates of ABCB1. Conversely, we found that DNA damaging drugs CPT and Ara-C were cytotoxic to taxanes-resistant cells. Treatment with either compound eradicated both the taxanes-resistant and taxanes-responsive (parental) cells at equivalent concentrations.

Camptothecin (CPT) is an alkaloid isolated from Chinese tree, Camptotheca acuminate [77]. This compound selectively inhibits Topoisomerase I, thus preventing DNA re-ligation during DNA replication, resulting in DNA damage and apoptosis. CPT analogues, Irinotecan and Topotecan, are FDA-approved chemotherapeutic agents [78] with wide range of antitumor effects in leukemia and in solid tumors (e.g., ovarian, breast, cervical, gastric, small cell lung). CPT is currently being evaluated in >150 clinical trials, including in 96 phase II and above (clinicaltrials.gov). Recently, a new CPT formulation as a nanoparticle-drug conjugate (NDC) of NLG207 (formerly CRLX101, currently EP0057) [79], was successfully tested in preclinical models of glioblastoma [80], triple-negative breast cancer [81], and advanced ovarian cancer [82]. In addition, EP0057 was successfully tested in a phase II clinical study for advanced ovarian [82], gastric and small cell lung cancers (clinicaltrials.gov). CPT and Irinotecan are structurally similar molecules [83], yet we observed that the latter is a substrate for ABCB1, while the former is not. Since RC4-2B cells have resistance to Irinotecan, the next step would be to include additional CPT-derived molecules in the screen with parental and taxane-resistant cell lines with the hope of identifying CPT derivatives that have the same effect in taxane-responsive and -resistant cells.

Treatment with a deoxycytidine nucleoside analog (1-β-D-arabinofuranosylcytosine) cytarabine (Ara-C, cytosine arabinoside) induces DNA damage and apoptosis. Ara-C is FDA approved and is among the most effective antineoplastic agents for treatment of variety of leukemias and Hodgkin and Non-Hodgkin lymphomas [84]. It is currently being tested in ~1500 clinical trials for a variety of cancers (including solid tumors), with >300 in phase III and IV (clinicaltrials.gov).

Although there are a few clinical trials that combine DTX with CPT (four trials) or Ara-C (three trials), none of them are designed to specifically target taxanes resistance in mCRPC (clinicaltrials.gov). We observed that treatment with either compound eradicated the taxanes-resistant cells at equivalent concentrations as those for the taxanes-responsive (parental) cells. Our results suggest a new treatment paradigm for patients diagnosed with terminal mCRPC. Our findings indicate that CPT or Ara-C treatment of taxanes-resistant tumors (at least those that overexpress ABCB1) can overcome this resistance, while combined treatment of taxanes and CPT or Ara-C is expected to eliminate acquired taxanes resistance. Our data show that antitumor drugs CPT or Ara-C are cytotoxic to mCRPC cells that overexpress ABCB1 and have acquired CBZ resistance, thus supporting this concept. Our results indicate a potential critical benefit of the combined treatment compared to the current standard of care using taxanes monotherapy.

CDK4 and CDK6 form a complex with Cyclin D to phosphorylate Rb protein, thus resolving G1/S checkpoint [60]. By inactivating Cyclin D-CDK4/6 complexes, CDK4/6 inhibitors prevent Rb phosphorylation, block release of E2F from Rb binding and inhibit cell entry into S phase. Being cytostatic, these inhibitors are used in combination with other classes of chemotherapies, as taxanes and DNA damage agents (that are cytotoxic) with hope to potentiate their activity as was recently demonstrated in pancreatic ductal adenocarcinoma cell models [85]. We found that CDK4/6i Palbociclib, Ribociclib and Abemaciclib have similar activity in parental and resistant CRPC cells, indicating that these CDK4/6i were not substrates of ABCB1. CDK4/6i Ribociclib potentiates activity of Ara-C and CPT in both parental and taxanes-resistant PCa cells. Moreover, we observed that treatment by DNA damaging agents followed by CDK4/6i reduced colonies growth stronger compared to the opposite regimen. Simultaneous treatment by CPT and Ribociclib does not affect CPT-induced apoptosis as tested by PARP-1 cleavage (Supplementary Fig. S6). It was demonstrated that long treatment (7 days) with CDK4/6 inhibitor Palbociclib induced senescence [85]. Thus, the most obvious explanation of our results is that inhibition of CDK4/6 blocks cells at the G1/S border, thus preventing activity of DNA damaging agents during S-phase, while the reversed treatment induces DNA damage followed by inhibition of cell cycle progression and activation of senescence of cells that escape DNA damage induced apoptosis. Thus, the most appropriate sequence of treatment should include Ara-C followed by CDK4/6i as Ribociclib.

**Fig. 8:**
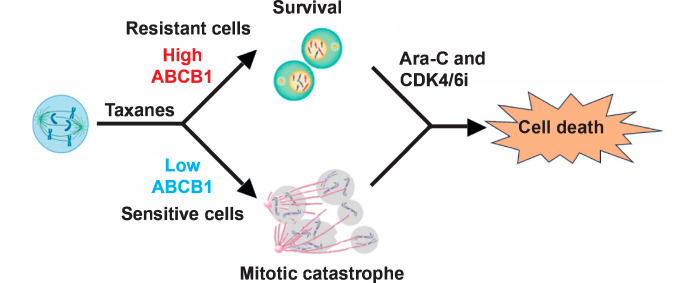
Model. Taxanes CBZ or DTX induce mitotic block in cells with low ABCB1 levels; they exit mitotic block by mitotic catastrophe and eventually die. Cells with high levels of ABCB1 are resistant to taxanes and complete mitosis. Treatment with Ara-C followed by CDK4/6i induces cell death, thus bypassing taxanes resistance.

In summary, our findings have identified DNA damage agents CPT and Ara-C as a potential treatment option in CRPC patients who have developed taxanes resistance due to overexpression of MDRP ABCB1. In addition, we found that sequential application of Ara-C followed by CDK4/6i the best among all the tested treatment combinations, suggesting a new treatment regimen in CRPC patients, including those that are resistant to taxanes treatment (Fig. 8). We predict that combined treatment with DNA damaging agents (as CPT or Ara-C) and CDK4/6i will eradicate CRPC cells, including subpopulation that acquired ABCB1 overexpression, therefore preventing acquired taxanes resistance.

### Supplementary information

Supplementary Materials and Figure Legends

Original data

## Data Availability

The datasets generated and analyzed during the current study are available in the NCBI Gene Expression Omnibus repository in series: GSE271075 (RNA-seq).
