# Supplementary material for: Overcoming ABCB1 mediated multidrug resistance in castration resistant prostate cancer
Source: Cell Death Dis. 2024 Aug 1;15(8):558. doi: 10.1038/s41419-024-06949-3 (PMC11294535; doi:10.1038/s41419-024-06949-3)
Supplement: Supplementary file 1 — Supplementary Materials and Figure Legends [file 41419_2024_6949_MOESM1_ESM.pdf]

**Supplementary Figure Legends**

# Overcoming ABCB1 mediated multidrug resistance in castration resistant prostate cancer

Sadia Sarwar[1*], Viacheslav M. Morozov[1*], Mallory A. Newcomb[1], Bowen Yan[3], Jason O. Brant[2,4], Rene Opavsky[1,4], Olga A. Guryanova[3,4], and Alexander M. Ishov[1,4]

*These authors contributed equally to this work

[1]Department of Anatomy and Cell Biology, University of Florida College of Medicine;

[2]Department of Biostatistics, University of Florida College of Medicine;

[3]Department of Pharmacology and Therapeutics, University of Florida College of Medicine;

[4]University of Florida Health Cancer Center, Gainesville, FL.

Corresponding author: Alexander M. Ishov, 2033 Mowry Road, Room 358, Gainesville FL 32610 tel: (352) 273-8202. E-mail: ishov@ufl.edu

**Fig. S1. Test of DNA damage drugs CPT, Ara-C and Irinotecan effect on parental and taxanes resistant cells.** C4-2B (parental) and RC4-2B CBZ-resistant cells were set up in Matrigel and treated with indicated concentrations of doxorubicin (**A**), CPT (**B**) and Ara-C (**C**) three days later for 24 h. On day 21, prostaspheres were stained with cell viability marker Calcein AM and documented. **Left:** representative images of prostasphere after treatment. **Right:** equal volumes of cells from trypsinized prostaspheres were re-plated in six well plates in triplicates and colonies were documented three weeks later. Representative images (**A, B, C**, **right)**; area of colonies was calculated with ImageJ (**D**). **E:** C4-2B and RC4-2B cells were treated with indicated concentrations of Irinotecan for 24 h; colonies were documented one week later. CNT: control.

**Fig. S2. ABCB1-specific inhibitor elacridar reverses CBZ and DTX resistance in taxanes resistant CRPC cells.** C4-2B and DU145 (parental), TaxR (C4-2B derived DTX resistant), DTXR (DU145 DTX resistant), and CTXR (DTXR derived DTX/CBZ resistant) cells were treated with indicated concentrations of CBZ or DTX alone, in combination with 0.5 uM of specific ABCB1i elacridar, or with 0.5 uM of

elacridar alone. After 72 h, viability was measured by Alamar Blue assay. Inhibition of ABCB1 reversed resistance to both taxanes (CBZ and DTX) in all resistant cells. CNT: control.

**Fig. S3. Characterization of CDK4/6i activity in CRPC cells.** Parental C4-2B and taxanes resistant RC4-2B cells were treated with indicated concentrations of CDK4/6i Abemaciclib (top), Palbociclib (middle), or Ribociclib (bottom) for 7 days. CNT: control.

**Fig. S4. Characterization of CDK4/6i Ribociclib (Ribo) activity.** Parental C4-2B and CBZ resistant RC4-2B cells were treated with the indicated concentrations of Ribo for 24h; CNT: control. **A, top and bottom:** analysis of cell cycle stages by FACS. % of G 1 was increased while % S and G2/M-phase cells was reduced in both cell lines after Ribo treatment. **B:** immunoblot characterization of **Left:** pRb phosphorylated at serines 807 and 811 (substrates of CDK4/6; rabbit ab's), and **Right:** same membrane probed for total pRb (mouse ab's ). pRbS807/8ll was reduced in both cell lines after Ribo treatment. Total pRb was reduced by Ribo treatment as previously published. ACTIN (mouse ab's): loading control.

**Fig. S5. Analysis of ABCB1 expression by Taxane exposure or by therapy outcome in PC patients. Left panel:** boxplots depicting mRNA FPKM values for ABCB1 expression (Y axis) in patients based on Taxane exposure status (X axis: exposed, Naïve, or unknown exposure status [UNK]). **Right panel:** boxplots depicting mRNA RSEM counts for ABCB1 expression (Y axis) for patients based on therapy outcome (X axis: complete response, partial response, or progressive disease). The number of patients in each group are listed at the top of the plot, and the mean expression value is listed at the bottom.

**Fig. S6. Western blot analysis of PARP-1 cleavage in C4-2B and RC4-2B cells after Ribociclib and CPT treatment.** In both cell lines, CPT (2uM) induces while Ribociclib (Ribo, 1uM) does not induce PARP-1 cleavage (marker of apoptosis). In combined treatment, Ribociclib does not affect CPT-induced PARP-1 cleavage. Actin: loading control. CNT: control.

**Fig. S7. Full immunoblot scans** corresponding to Fig. 3E (top) and Fig. 5C (bottom).

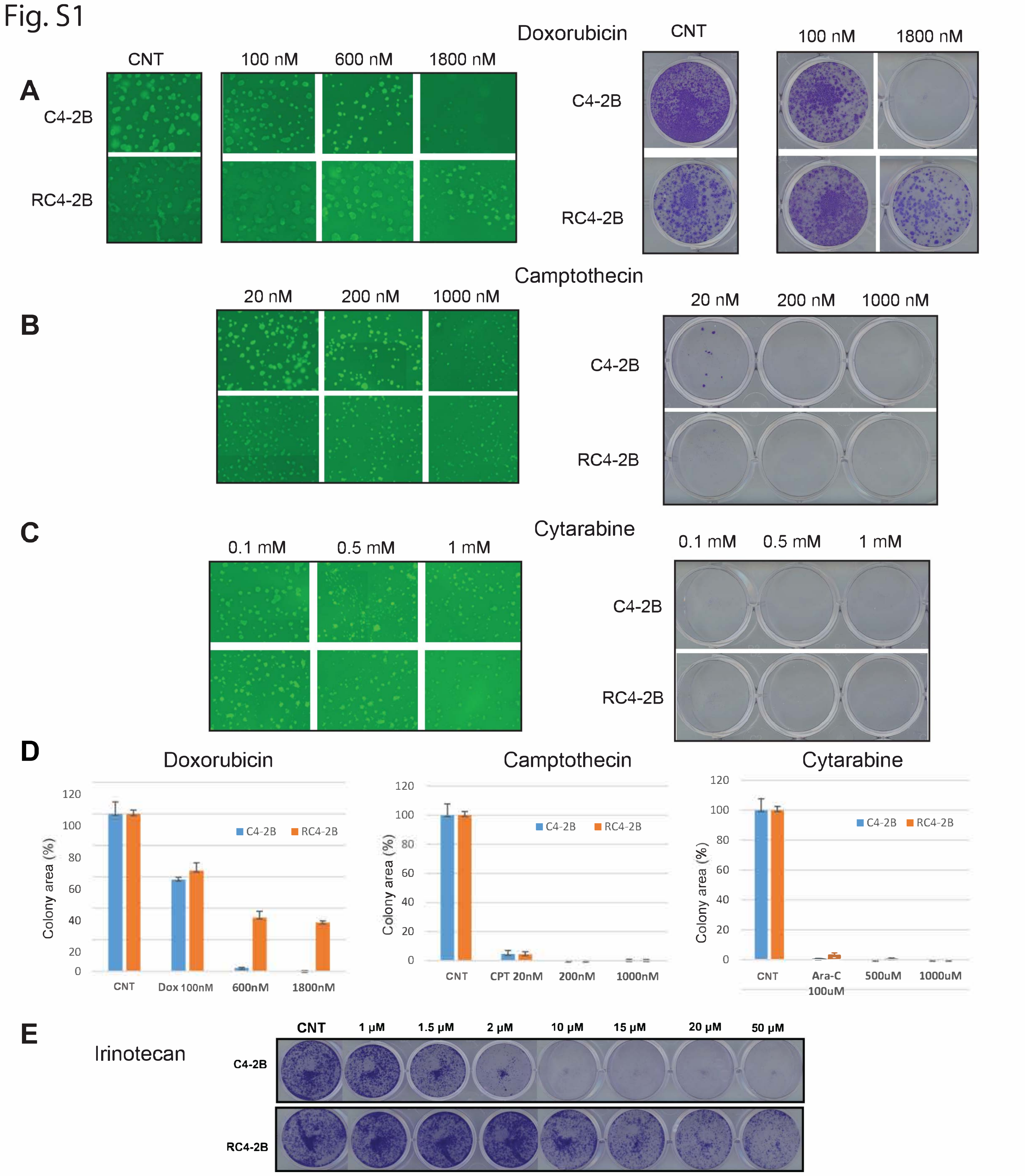


**Fig. S1. Test of DNA damage drugs CPT, Ara-C and Irinotecan effect on parental and taxanes resistant cells.** C4-2B (parental) and RC4-2B CBZ-resistant cells were set up in Matrigel and treated with indicated concentrations of doxorubicin (**A**), CPT (**B**) and Ara-C (**C**) three days later for 24h. On day 21, prosta-spheres were stained with cell viability marker Calcein AM and documented. **Left:** representative images of prostasphere after treatment. **Right:** equal volumes of cells from trypsinized prostaspheres were re-plated in six well plates in triplicates and colonies were documented four weeks later. Representative images (**A, B, C, right**); area of colonies was calculated with ImageJ (**D**). **E:** C4-2B and RC4-2B cells were treated with indicated concentrations of Irinotecan for 24h; colonies were documented one week later. IC50 is ~10x higher in RC4-2B compared to C4-2B cells. CNT: control.

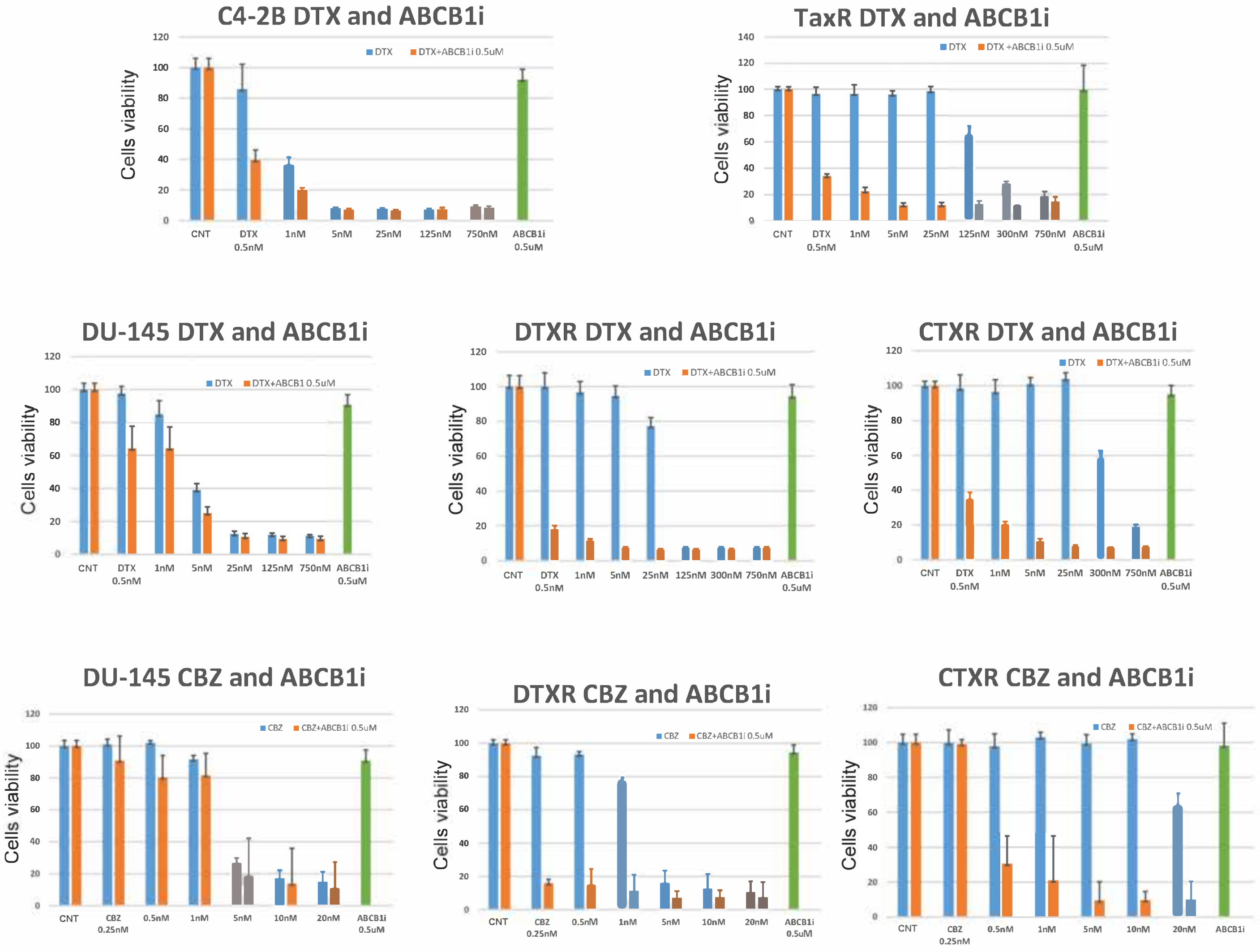


**Fig. S2. ABCB1-specific inhibitor elacridar reverses CBZ and DTX resistance in taxanes resistant CRPC cells.** C4-2B and DU145 (parental), TaxR (C4-2B derived DTX resistant), DTXR (DU145 DTX resistant), and CTXR (DTXR derived DTX/CBZ resistant) cells were treated with indicated concentrations of CBZ or DTX alone, in combination with 0.5 uM of specific ABCB1i elacridar, or with 0.5 uM of elacridar alone. After 72h, viability was measured by Alamar Blue assay. Inhibition of ABCB1 reversed resistance to both taxanes in all resistant cells. CNT: control.

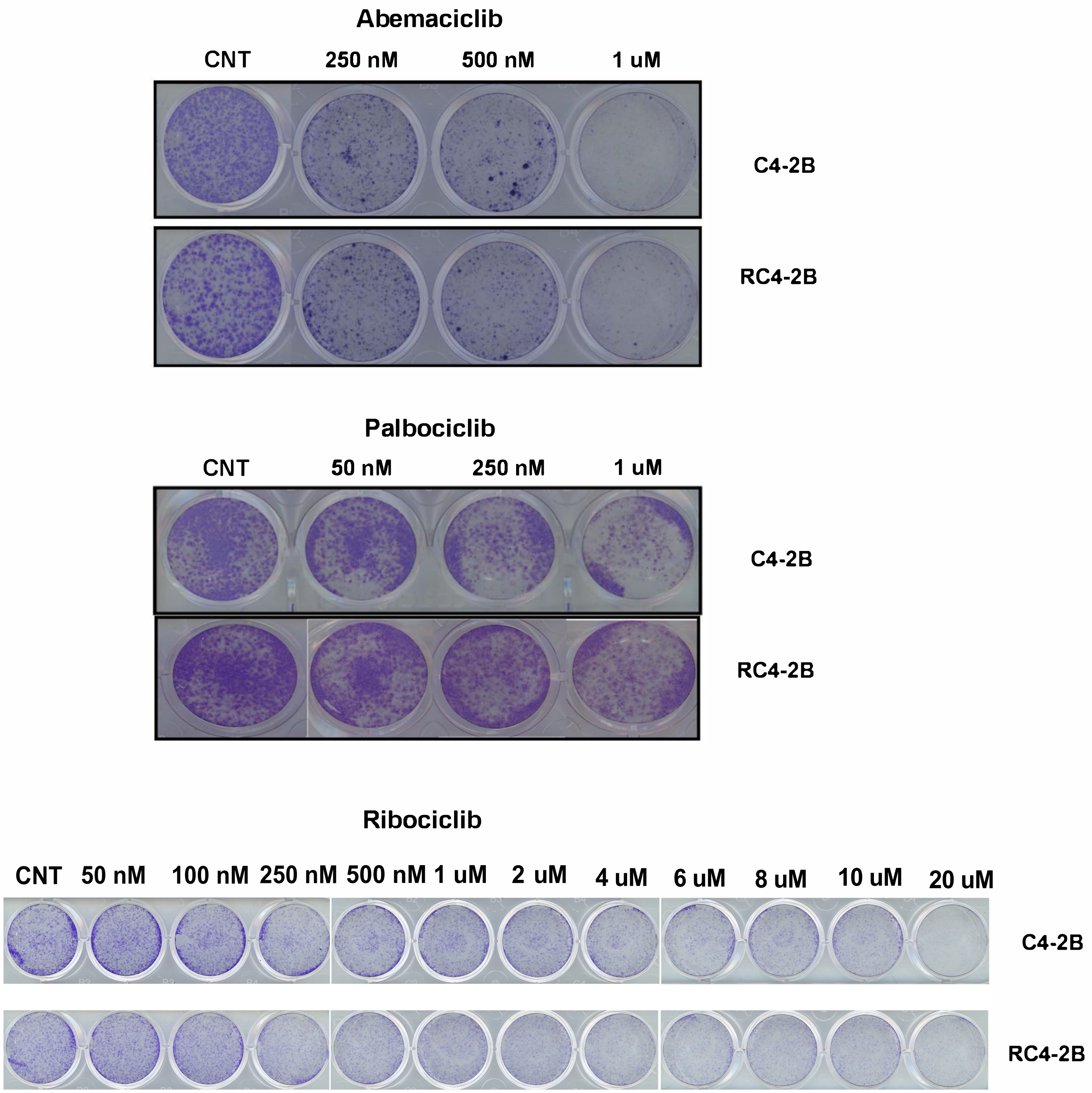


**Fig. S3. Characterization of CDK4/6i activity in CRPC cells.** Parental C4-2B and taxane resistant RC4-2B cells were treated with indicated concentrations of CDK4/6i Abemaciclib (top), Palbociclib (middle), or Ribociclib (bottom) for 7 days. CNT: control.

**A**

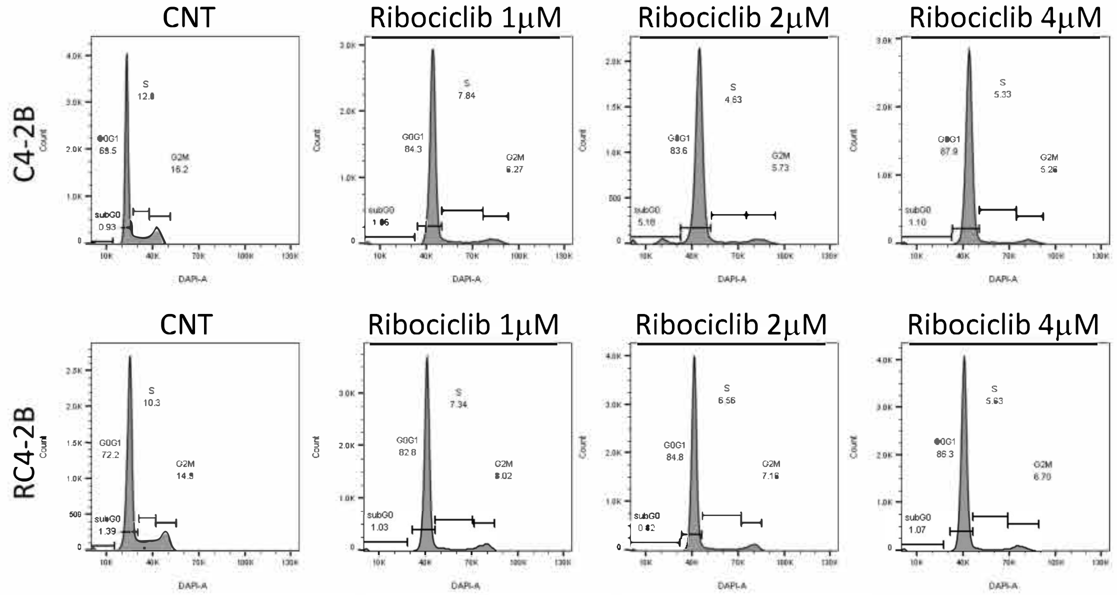


| | G1 | G2/M | S | sub-G1 |
|---|---|---|---|---|
| **C4-2B** | | | | |
| CNT | 69.5 % | 16.2 % | 12.0 % | 0.93 % |
| Ribociclib 1 uM | 84.3 % | 6.27 % | 7.84 % | 1.06 % |
| Ribociclib 2 uM | 83.6 % | 5.73 % | 4.63 % | 5.18 % |
| Ribociclib 4 uM | 87.9 % | 5.26 % | 5.33 % | 1.10 % |
| | | | | |
| **RC4-2B** | | | | |
| CNT | 72.2 % | 14.9 % | 10.3 % | 1.39 % |
| Ribociclib 1 uM | 82.8 % | 8.02 % | 7.34 % | 1.03 % |
| Ribociclib 2 uM | 84.8 % | 7.16 % | 6.56 % | 0.82 % |
| Ribociclib 4 uM | 86.3 % | 6.70 % | 5.63 % | 1.07 % |

**B**

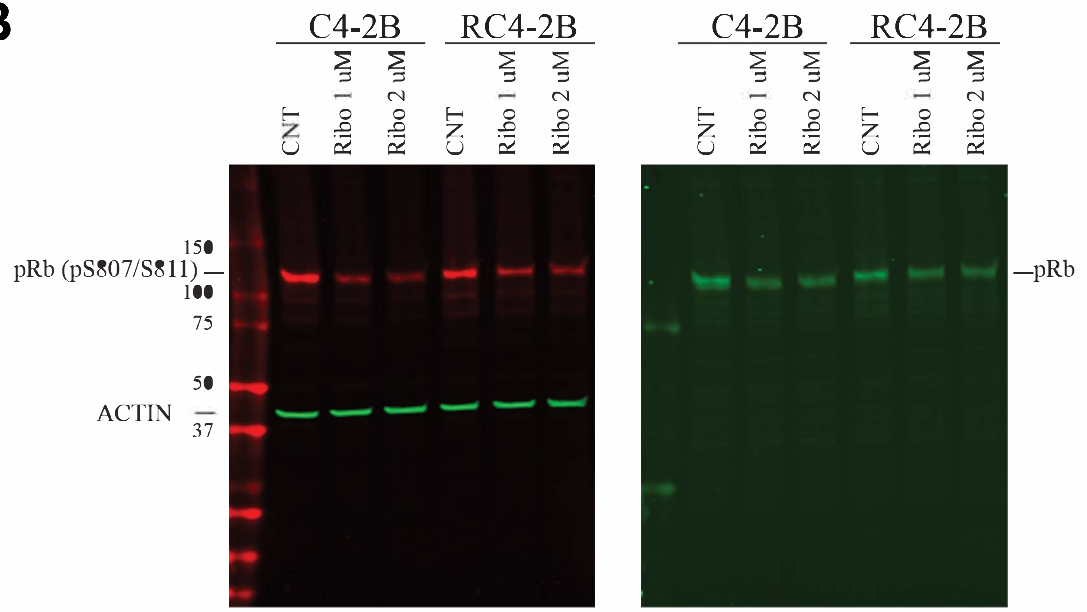


**Fig. S4. Characterization of CDK4/6i Ribociclib (Ribo) activity.** Parental C4-2B and CBZ resistant RC4-2B cells were treated with the indicated concentrations of Ribo for 24h; CNT: control. **A, top and bottom:** analysis of cell cycle stages by FACS. % of G1 was increased while % S and G2/M-phase cells was reduced in both cell lines after Ribo treatment. **B:** immunoblot characterization of **Left:** pRb phosphorylated at serines 807 and 811 (substrates of CDK4/6; rabbit ab's), and **Right:** same membrane probed for total pRb (mouse ab's) pRbS807/811 was reduced in both cell lines after Ribo treatment. Total pRb was reduced by Ribo treatment as previously published. ACTIN (mouse ab's): loading control.

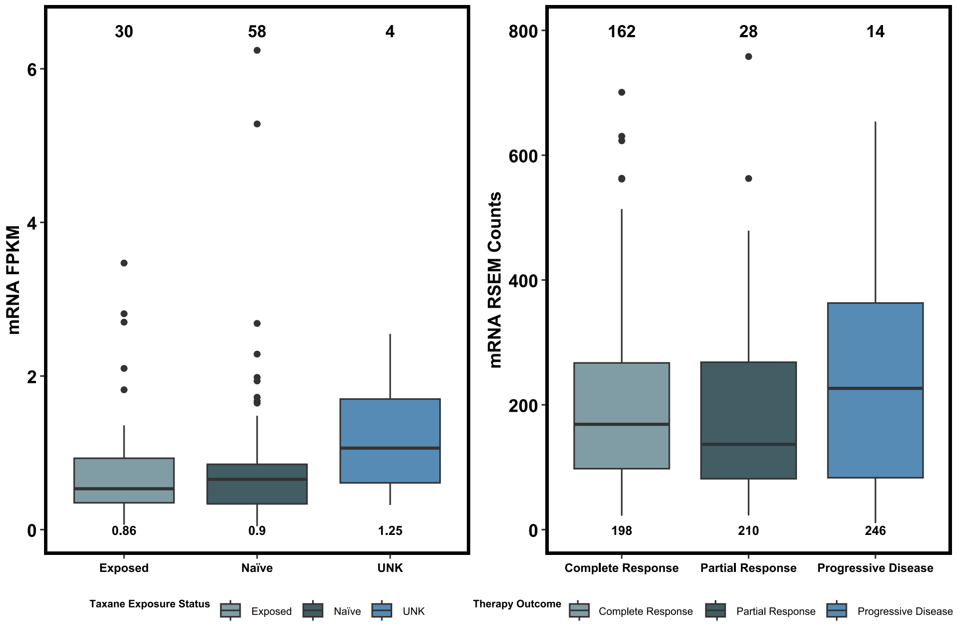


**Fig. S5. Analysis of ABCB1 expression by Taxane exposure or by therapy outcome in PC patients. Left panel:** boxplots depicting mRNA FPKM values for ABCB1 expression (Y axis) in patients based on Taxane exposure status (X axis: exposed, Naïve, or unknown exposure status (UNK)). **Right panel:** boxplots depicting mRNA RSEM counts for ABCB1 expression (Y axis) for patients based on therapy outcome (X axis: complete response, partial response, or progressive disease). The number of patients in each group are listed at the top of the plot, and the mean expression value is listed at the bottom.

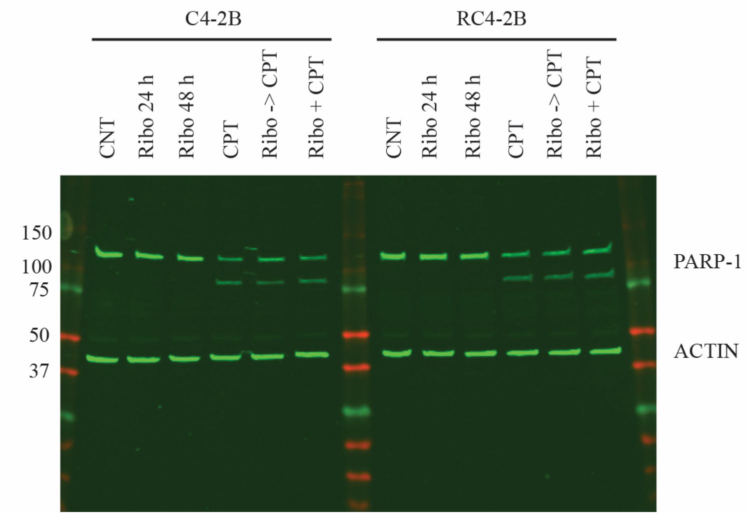


**Fig. S6. Western blot analysis of PARP-1 cleavage in C4-2B and RC4-2B cells after Ribociclib and CPT treatment.** In both cell lines, CPT (2uM) induces while Ribociclib (Ribo, 1uM) does not induce PARP-1 cleavage (marker of apoptosis). In combined treatment, Ribociclib does not affect CPT-induced PARP-1 cleavage. Actin: loading control. CNT: control.

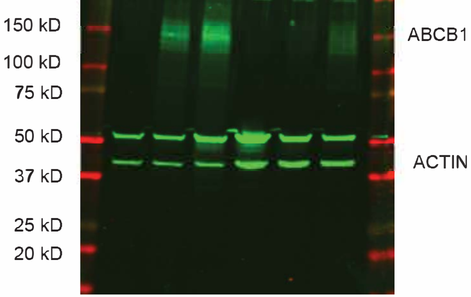


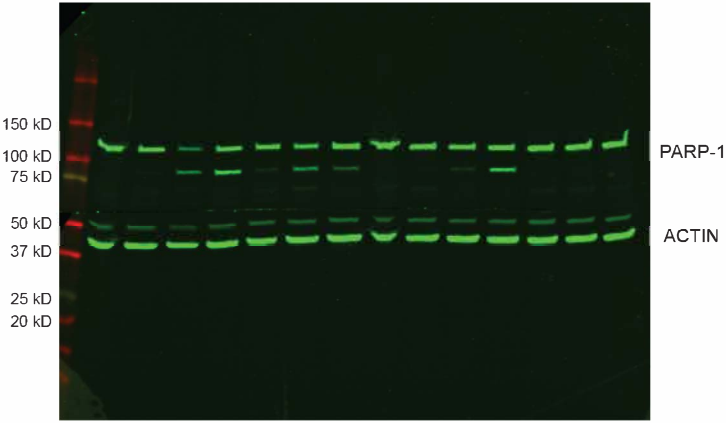


**Fig. S7.** Full scan of gels for Fig. 3E (top) and Fig. 5C (bottom).
