# Supplementary figures and images for: Overcoming ABCB1 mediated multidrug resistance in castration resistant prostate cancer
Source: Cell Death Dis. 2024 Aug 1;15(8):558. doi: 10.1038/s41419-024-06949-3 (PMC11294535; doi:10.1038/s41419-024-06949-3)

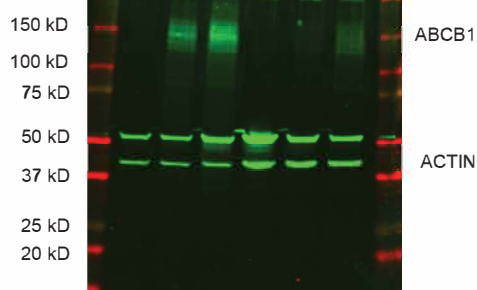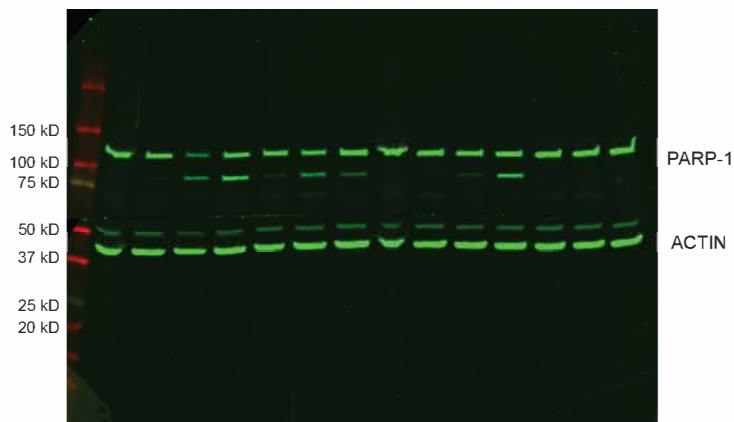

**Fig. S7.** Full scan of gels for Fig. 3E (top) and Fig. 5C (bottom).

Supplement: Supplementary file 2 — Original data [file 41419_2024_6949_MOESM2_ESM.pdf]
